# Improving the Modeling of Disease Data from the Government Surveillance System: A Case Study on Malaria in the Brazilian Amazon

**DOI:** 10.1371/journal.pcbi.1003312

**Published:** 2013-11-07

**Authors:** Denis Valle, James Clark

**Affiliations:** 1 School of Forest Resources and Conservation, Tropical Conservation and Development Program (Center for Latin American Studies), Emerging Pathogens Institute, University of Florida, Gainesville, Florida, United States of America; 2 Nicholas School of the Environment, Department of Biology, Department of Statistical Science, Duke University, Durham, North Carolina, United States of America; Imperial College London, United Kingdom

## Abstract

The study of the effect of large-scale drivers (e.g., climate) of human diseases typically relies on aggregate disease data collected by the government surveillance network. The usual approach to analyze these data, however, often ignores a) changes in the total number of individuals examined, b) the bias towards symptomatic individuals in routine government surveillance, and; c) the influence that observations can have on disease dynamics. Here, we highlight the consequences of ignoring the problems listed above and develop a novel modeling framework to circumvent them, which is illustrated using simulations and real malaria data. Our simulations reveal that trends in the number of disease cases do not necessarily imply similar trends in infection prevalence or incidence, due to the strong influence of concurrent changes in sampling effort. We also show that ignoring decreases in the pool of infected individuals due to the treatment of part of these individuals can hamper reliable inference on infection incidence. We propose a model that avoids these problems, being a compromise between phenomenological statistical models and mechanistic disease dynamics models; in particular, a cross-validation exercise reveals that it has better out-of-sample predictive performance than both of these alternative models. Our case study in the Brazilian Amazon reveals that infection prevalence was high in 2004–2008 (prevalence of 4% with 95% CI of 3–5%), with outbreaks (prevalence up to 18%) occurring during the dry season of the year. After this period, infection prevalence decreased substantially (0.9% with 95% CI of 0.8–1.1%), which is due to a large reduction in infection incidence (i.e., incidence in 2008–2010 was approximately one fifth of the incidence in 2004–2008).We believe that our approach to modeling government surveillance disease data will be useful to advance current understanding of large-scale drivers of several diseases.

## Author Summary

Disease data collected by the government surveillance system are frequently used to understand the influence of large-scale phenomena (e.g., climate) on human health because these data often have a large temporal and/or geographical span. The down side is that a) these data are often biased towards individuals that come to the health facilities (i.e., symptomatic individuals); and b) the number of individuals examined can vary substantially regardless of concurrent changes in prevalence or incidence (e.g., due to shortage of personnel or supplies in health facilities), directly impacting the number of disease cases detected. Current modeling approaches typically ignore these peculiarities of the government data. Furthermore, current approaches do not take into account that observations directly influence disease dynamics since individuals with a positive diagnosis are often subsequently treated for the disease. In this article, we develop a novel model to circumvent these shortcomings and apply it to simulated data, highlighting how inference on infection incidence and prevalence might be misleading when some of the issues mentioned above are ignored. Finally, we illustrate this model using malaria data from the Brazilian Amazon, revealing the strong role of precipitation on infection prevalence seasonality and striking patterns in infection incidence.

## Introduction

Current best practices regarding the collection of disease data consist in the unbiased sampling of individuals (e.g., through aggressive active case detection; [1], [2]) using the most sensitive pathogen detection method available (e.g., polymerase chain reaction (PCR) for malaria). This type of individual-level data has provided important information regarding infection and disease (symptoms+infection) prevalence and risk factors; however, these data are costly and thus tend to be spatially and temporally restricted, curtailing their ability to detect important disease drivers that vary over long temporal and large spatial scales. Studies that focus on large geographical and/or long temporal-scale disease drivers typically rely on government-based surveillance data (e.g., malaria [3]–[6], cholera [7], [8], measles [9], [10], american cutaneous leishmaniasis [11], pertussis [12], meningitis [13], and dengue [14]). While government-based surveillance data provide a wealth of information on disease, these data are often collected opportunistically, which may severely bias inference drawn from these data [e.g.], [ 15,16]. For instance, individuals routinely sampled by the government health facilities are often symptomatic [17], [18]. As a result, if part of the population is infected but asymptomatic, infection prevalence for the overall population cannot be estimated as if these data came from a random sample (i.e., the number detected to be infected divided by number of tested individuals) nor as if all infected individuals had been detected (i.e., the number detected to be infected divided by total population size). Similarly, the number of individuals that seek help at a particular health facility may fluctuate considerably with time regardless of concurrent changes in infection prevalence or incidence (e.g., due to increases in catchment area, or a shortage of personnel or supplies), directly affecting the number of observed disease cases. Unfortunately, past analyses have typically considered only the number of disease cases per unit time (e.g., weekly or monthly), ignoring the total number of individuals examined per unit time (but see [19]).

The standard approach to analyze time-series data from the government surveillance system is to search for trends [e.g.], [ regression analysis]; [ 3], [4], [11], [20]–[23] or scales of variability [e.g.], [ wavelet analysis]; [ 10], [24–26] that match those of the explanatory variables. Recent work, however, has increasingly employed sophisticated statistical models, typically within the state-space modeling framework, to fit mechanistic disease dynamics models [e.g.], [ 7], [9], [27]–[32]. An important assumption within these state-space models is that observations provide information about the states but do not affect the underlying process. In the particular context of disease dynamics, the assumption is that the number of individuals diagnosed with a particular disease provides information on infection incidence or prevalence but does not influence disease dynamics (the underlying temporal process). This is a valid assumption if tested individuals are not informed about test results nor treated for the disease (e.g., data consist on the number of deaths due to a particular disease). However, this assumption is violated if individuals that have a positive diagnosis are subsequently treated for the disease because treatment decreases the pool of infected individuals and thus affects disease dynamics.

Here we refine the state-space framework to overcome the shortcomings we have described. Our approach scales-up the results from a detailed individual-level study to allow unbiased inference on infection prevalence from government-based syndromic surveillance data over larger geographical and longer temporal scales than would be possible using solely the individual-level data. Our approach also properly accounts for changes in sampling effort and the number of individuals diagnosed/treated for the disease and makes use of several short time-series (rather than one long time-series) to infer changes in infection prevalence and the drivers of these changes. While some of our assumptions are tailored to malaria, the general approach we put forth should be adaptable to other human diseases.

We start our article by describing our data and the model we are proposing. We then use a ten-fold cross-validation exercise to show that the proposed model has a better out-of-sample predictive performance than a more phenomenological statistical model and a more mechanistic disease dynamics model. Next, we employ simulated data to show how inference on disease incidence can be severely distorted if one does not take into account concurrent changes in sampling effort and that observations affect disease dynamics. Finally, we illustrate our model by applying it to real malaria data from the western Brazilian Amazon.

## Methods

### Data

**Figure 1:**
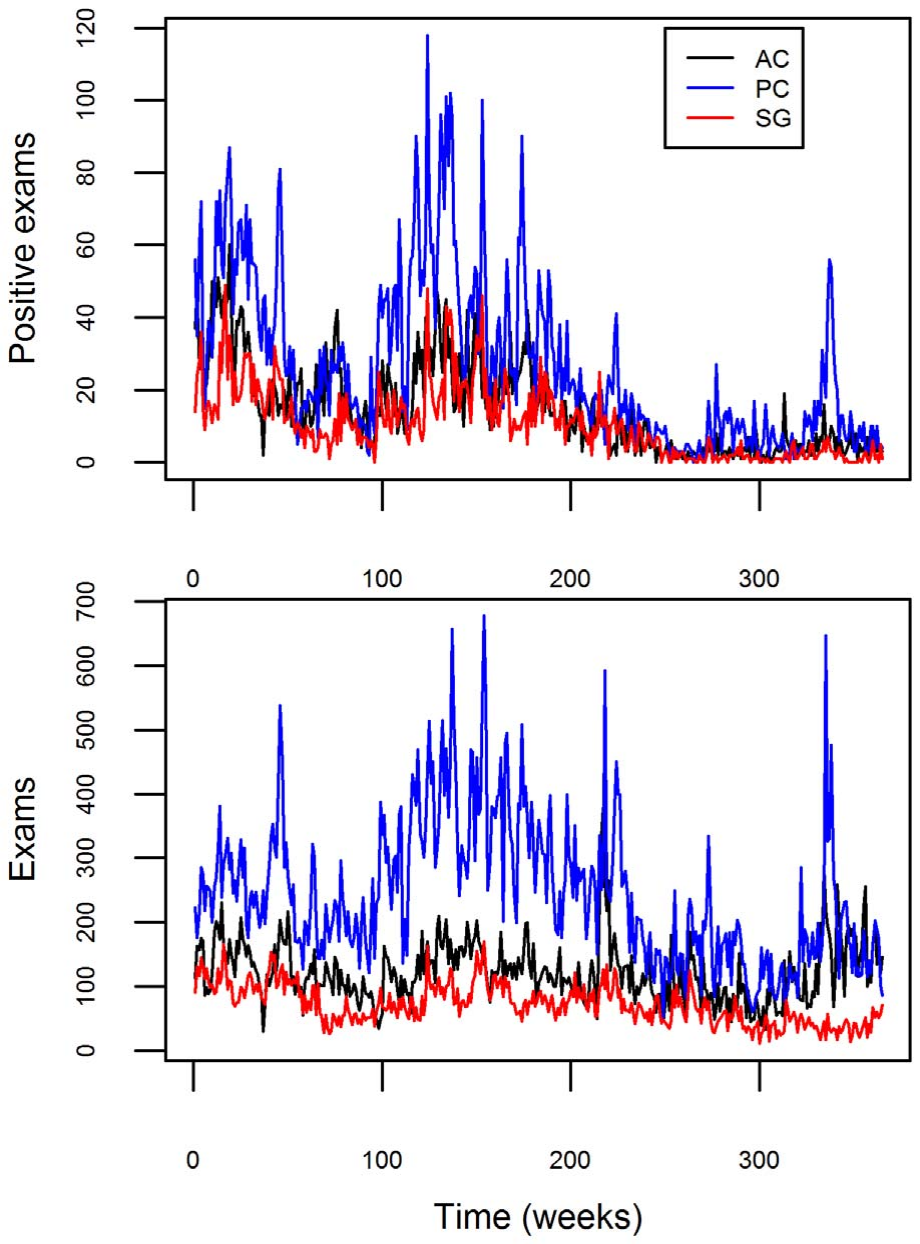
Temporal and geographical distribution of the government surveillance malaria data. Malaria data depiction for Acrelandia (AC), Placido de Castro (PC), and Senador Guiomard (SG) counties (black, blue, and red lines, respectively). Number of positive exams and total number of exams are shown in upper and lower panels, respectively.

Malaria health posts are the only source of antimalarial medication in the Brazilian Amazon and this medication can only be obtained with a positive malaria exam result. As a result, data from these health posts provide considerable information regarding changes in malaria prevalence and incidence, being the basis of the malaria surveillance system in Brazil [33]. The malaria data we use arise from the Brazilian surveillance network in three counties (Acrelandia – AC, Placido de Castro – PC, and Senador Guiomard – SG) in Acre state, western Brazilian Amazon. These data are aggregated by week *t* and county *l*. Over the entire 2004–2010 period, there were approximately 160,000 malaria tests, from which ∼20,000 were positive (Figure 1). In this dataset, individuals are sampled and tested for malaria (through microscopy) either because they believed they had malaria and sought help at the local government health facility (passive case detection) or because they were symptomatic when health agents visited their houses (active case detection). In either case, individuals tend to be predominantly symptomatic.

### Model description

#### Observation model: scaling up individual-level data

The standard observation model in disease dynamics models assumes that the observed number of new cases is proportional to the true number of new infections (infection incidence) [7], [9], [27]–[29], [31], [32], an assumption that may not be realistic. Reasons for failing to detect these infected individuals when they first become infected include these individuals being a) originally asymptomatic and thus not sampled by the health facility; b) symptomatic but also not sampled by the health facility (e.g., due to access issues in reaching the health facility); or c) symptomatic, sampled by the health facility, but misdiagnosed with a negative exam result due to the low sensitivity of the diagnostic method. As a consequence, with few exceptions (e.g., an acute disease with a known and well-defined incubation period), the infection date of individuals is often highly uncertain [e.g., 16].

Here we adopt an alternative observation model which relates the observed number of malaria cases to the proportion of the population that is infected (infection prevalence), rather than infection incidence. But how can the information from the government surveillance data be related to the infection prevalence of the overall population if we know that the data are biased (i.e., most of the individuals sampled by health facilities tend to be symptomatic)? In the following sections we show how this can be done using an auxiliary unbiased dataset.

Let 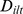 be the event of malaria detection for individual i sampled by the government surveillance system at county l (l = 1,2,3) and time t (t = 1,…,T). We start by assuming that 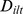 is a Bernoulli event with success probability
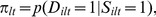
(1)where 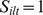 indicates that individual i at time t and county l was sampled by the government surveillance system. As a result of this assumption, the total number of positive tests in a given week and time 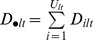
can be modeled as
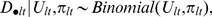
(2)where 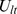 is the number of malaria tests.

Let infection status be denoted by 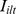. The main quantity we are interested in estimating is the number of infected individuals at time t and county l, 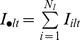, where 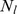 is the population size. Note that 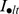 is the current number of infected individuals and not the number of newly infected individuals at time t (i.e., infection incidence). To relate this quantity to eqn. 1, we start by marginalizing over all possible symptomatic statuses 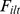 (which stands for fever) and infection statuses:
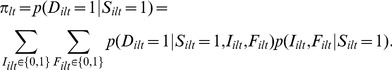
(3)


We simplify this expression by adopting several assumptions. We assume that only infected individuals can have a positive microscopy detection and that knowing that an individual was sampled by the government surveillance system does not influence the detection probability nor the probability of being infected given symptomatic status. These assumptions are formalized as:
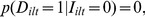
(A1)

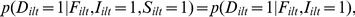
(A2)

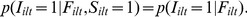
(A3)


Assumption A1 arises from the fact that it is unlikely that an experienced microscopist will identify malaria pathogens on a blood sample from an uninfected patient, regardless of the symptomatic status of the patient [Ferreira, personal communication; 34]. Indeed, using PCR as the reference test, data from the Brazilian Amazon consistently show a very low rate of false positives from microscopy: 0.78% (7/891) [35], 0% (0/214) [36], and 0.53% (6/1127) [37]. In relation to assumptions A2 and A3, because the main bias associated with the government surveillance data refers to the sampling of predominantly symptomatic individuals, if we condition on knowing the symptomatic status of the individual, then the fact that the individual was sampled should provide no further information regarding detection or infection probability.

As a result of these simplifying assumptions, eqn. 3 becomes
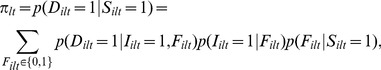
(4)which, using Bayes rule, can be expressed as
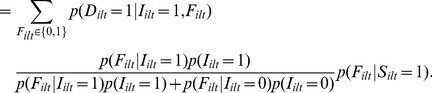
(5)


Here we assume that all probabilities in eqn. 5 are the same across individuals and that the conditional probabilities do not change over time or county. Thus, we will denote these conditional probabilities as parameters to be estimated:
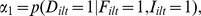


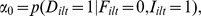


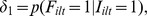


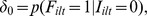


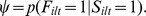



Furthermore, because population size 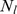 is large in each county (ranging from 11,000 to 19,000 people), we approximate 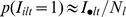. Using this notation, eqn. 5 becomes
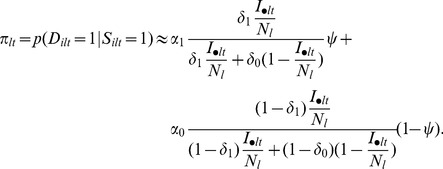
(6)


**Figure 2:**
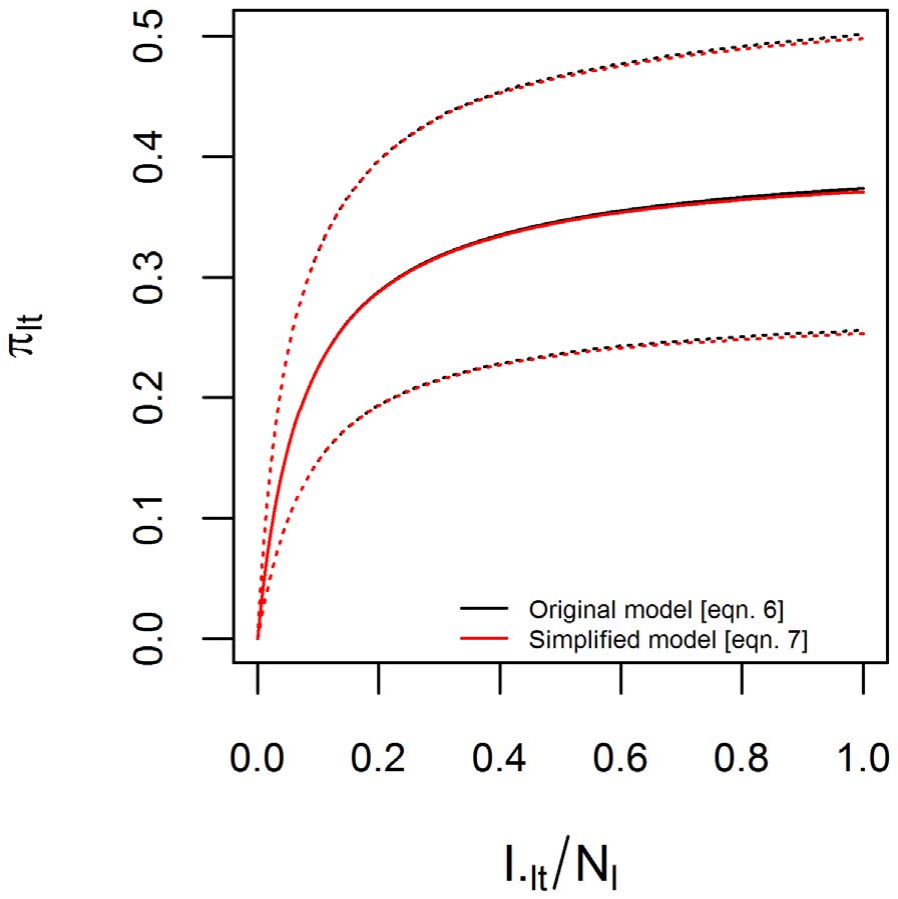
Prior relationship between detection probability given sampled and infection prevalence. Approximate relationship between detection probability given that the person was sampled by the government surveillance system 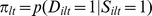 and infection prevalence 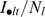, based on informative priors on the parameters of the observation model (Table 2). Solid and dashed lines are the median and 95% prior credible intervals based on the original (eqn. 6, black lines) and simplified (eqn. 7, red lines) observation models.

Finally, prior information (see “Prior Distributions” section) suggests that the second component of eqn. 6 is negligible (Figure 2), because both 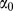 and 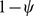 are small. The first parameter 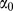 is small because microscopy has low sensitivity for individuals that do not have symptoms and 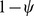 is small because it is rare for individuals without symptoms to be sampled by the government health facilities. As a result, we dropped the second component of eqn. 6, yielding
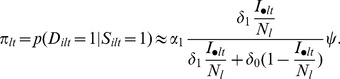
(7)


We note that the terms in eqn. 6 are clearly unidentifiable in the absence of prior information because we are estimating five fixed parameters (i.e., 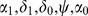) and one varying latent state (i.e., 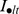) for every 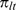. Furthermore, the simplification in eqn. 7 does not eliminate problems regarding parameter identifiability (e.g., note that 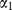 and 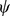 are still unidentifiable), indicating that prior information on these parameters will be critical to estimate model parameters and latent states (see “Prior distributions” section).

In summary, our observation model for the aggregate government surveillance data can succinctly be described as
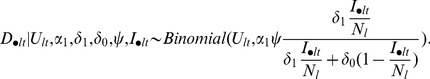
(8)


Notice that eqn. 7 implies a non-linear relationship between infection prevalence and detection probability given that the person was sampled (Figure 2). The intuition for this non-linear relationship is simple; when infection prevalence is low (i.e., 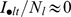), most of the symptomatic individuals that seek help at the government health facilities are uninfected, resulting in very low proportion of positive exams (i.e., 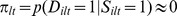). On the other hand, even if the entire population is infected (i.e., 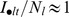), there is still an upper limit <1 to the proportion of positive exam results (i.e., 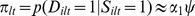).

#### Process model: describing the disease dynamics

Our process model describes how the number of infected individuals at time *t* and county *l* 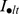 vary through time. While standard disease dynamics model often account for the number of individuals treated for the disease as an additional parameter to be estimated, in our case we know how many individuals were treated at each time. Thus, we expect that the number of infected individuals at time t+1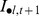 will be equal to the original number of infected individuals 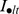 plus the number of newly infected individuals 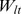 minus the infected individuals that were detected and cured 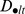 and the individuals that recover naturally from infection 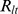 (we ignore people moving in and out of the county). This can be succinctly described as:
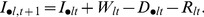
(9)


We are interested in assessing how environmental factors influence 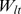. We start by noting that 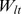 is not separately identifiable from 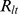 in our model since recovery from malaria is not a well-known and well-defined process; thus, we model changes in 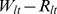. We refer to this quantity simply as infection incidence since we expect the number of recovered individuals 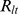 to be small relative to the number of newly infected individuals 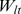. We replace 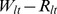 in equation 9 by its annual average 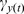 (the subscript y(t) denotes the year that includes week t, where y(t) = 1,…7). Preliminary analysis suggested that alternative parameterizations (e.g., adopting monthly averages or county specific yearly averages) resulted in poor convergence of the algorithm. Then, we assume that the expected number of prevalent infected individuals at time t+1 is given by:
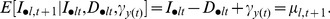
(10)


**Figure 3:**
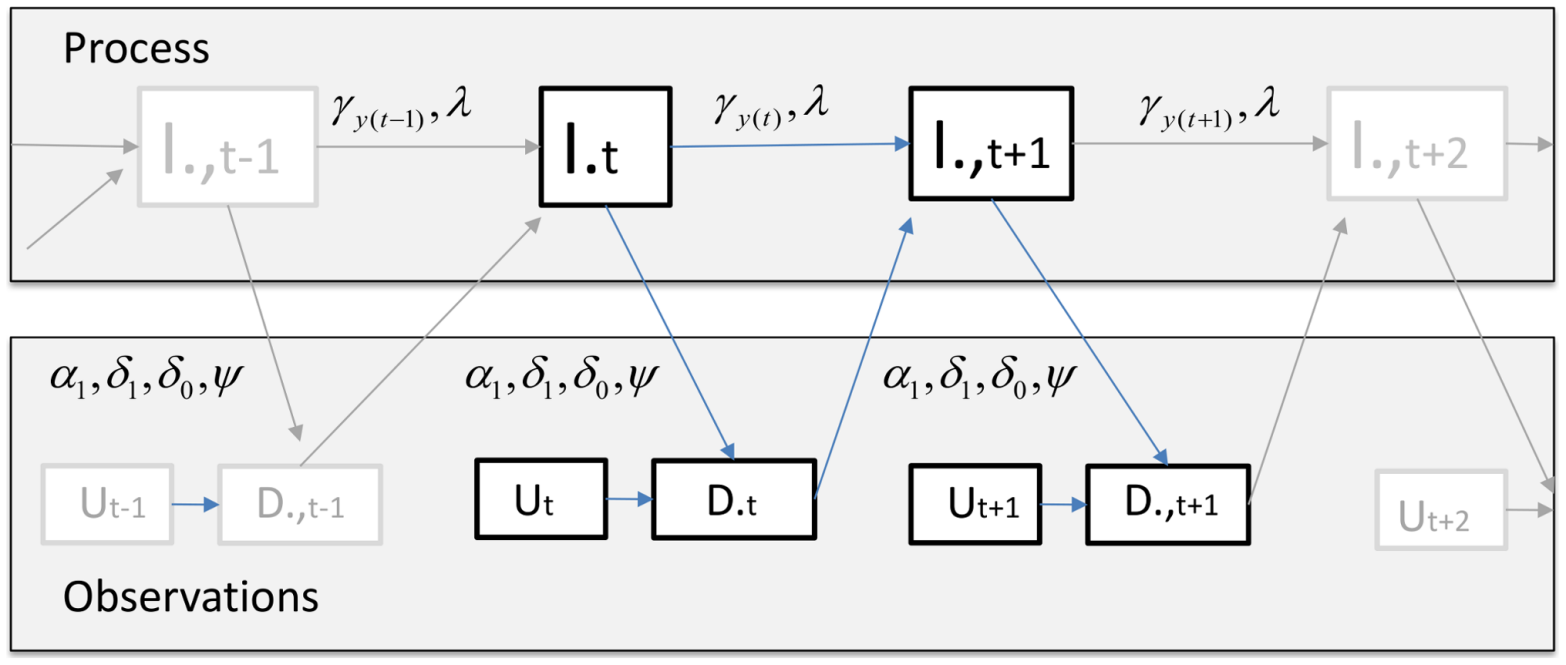
Model structure. The structure of the proposed model is depicted here for a given county l (we drop the county subscripts l to avoid clutter). 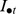 is the latent number of infected individuals at time t; 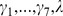 and 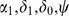 are parameters to be estimated; and 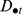 and 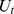 are the number of positive exams and total number of exams, respectively.

We allow for uncertainty in our process model (e.g., departures from equation 10 due to model misspecification and the approximation of a continuous phenomenon into a discrete one) by assuming that:
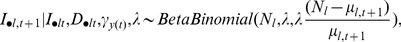
(11)where the beta-binomial distribution is such that if 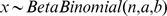 then 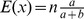. In eqn. 11, the expected value of 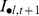 is given by eqn. 10 and extra-binomial variability is accounted for by the parameter 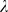. All these variables are summarized in Table 1 and the relationship between them is illustrated in Figure 3. This figure emphasizes the fact that observations directly impact infection prevalence dynamics through the treatment of individuals diagnosed to be infected, in sharp contrast with the usual assumption in state-space models.

**Table 1:** Summary of notation.

Event	Description
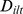	Malaria detection through microscopy
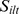	Sampled by the government surveillance system
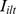	Infection status
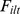	Symptom status
**Data**	
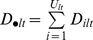	Total number of malaria cases detected
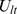	Total number of individuals examined for malaria
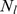	Population size
**Parameters**	
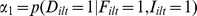	Microscopy sensitivity given symptoms
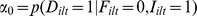	Microscopy sensitivity given lack of symptoms
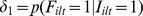	Probability of symptoms given infected
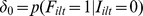	Probability of symptoms given not infected
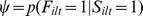	Probability of symptoms given sampled
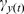	Annual mean of infection incidence
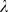	Extra-binomial variability parameter
**Latent states**	
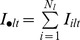	Total number of infected individuals

We could have adopted a more mechanistic representation of disease dynamics in eqn. 10. Indeed, several modelers have used a directly transmitted disease model for a vector-transmitted disease under the assumption that vector dynamics are fast relative to disease dynamics [e.g.], [ 38], [39]–[41]. For example, in a Susceptible-Infectious-Susceptible (SIS) framework, a typical assumption is that 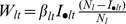, where 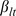 is the transmission rate (often modeled as a function of environmental covariates). However, we prefer the phenomenological formulation in eqn. 10 over a more mechanistic representation (e.g., SIS model) for several reasons. First, preliminary attempts to fit a SIS model while also allowing for process error revealed that several parameters were unidentifiable. This is not a feature unique to our model and/or disease data; parameters from biologically inspired disease dynamics model are notorious for having weakly identifiable parameters (e.g., hospital infections [42], Ebola Haemorrhagic Fever [43], malaria [28], [44], and influenza [45], [46]). Second, SIS or SEIR disease dynamic models have several simplifying assumptions of their own (e.g., approximating a mosquito transmitted disease with a direct transmission disease model, assuming homogeneous mixing, and exponentially distributed latent and infectious periods). In particular, these mechanistic models have substantial model structure uncertainty because alternative sets of simplifying assumptions can lead to dramatically different results [47], [48]. Yet, despite this model structure uncertainty, several modelers often assume that noise arises solely from measurement/observation error and that disease dynamics are perfectly described by the underlying model (i.e., no process error) [49]–[51]. Finally, a cross-validation exercise (described at a later section) revealed that the proposed model (eqns. 8 and 11) outperformed a deterministic SIS model.

#### Prior distributions

If all the terms in our observation model (eqn. 8) were unknown, it would be impossible to separately estimate them using just the government surveillance data. Intuitively it is clear why this ought to be the case; in the absence of additional information, it is impossible to estimate infection prevalence for the entire population just using data from predominantly symptomatic individuals. Thus, we relied on information from an auxiliary individual-level dataset collected within the study region to generate informative priors on some of these terms.

This auxiliary dataset was collected in a rural settlement area within Acrelandia on 486 individuals using four cross-sectional surveys (March/April 2004, September/October 2004, February/March 2005, and October/November 2006; all consenting study participants that were present at the time of the survey were sampled, regardless of their symptomatic status) and by searching for malaria exam results on the same set of individuals at the local health facility records. These data contained a total of 3,077 microscopy and 1,400 PCR malaria tests. We assume these data to be representative because a) they were collected within the same region and time frame that we are studying; b) the age structure of the sampled individuals is similar to the age structure of the overall population in these counties; c) most of the area of these three counties is covered by similar rural settlements; and d) there was a strong correlation (0.65) between the time series of malaria cases from this detailed study and the time series at the county level. Further details on the area, data collection, and characteristics of the study participants can be found elsewhere [37], [52]–[54].

We model this individual level dataset with a binomial likelihood, assuming PCR as the reference test. As result, we can summarize the information in this dataset by calculating the number of successes and total number of observations (i.e., trials) related to each parameter in our observation model (“Prior data” column in Table 2). Using the number of successes and observations and assuming a uniform prior distribution, we can obtain a posterior beta distribution with parameters a and b (“Posterior Beta distribution” column in Table 2). We use these beta distributions as informative priors for our study. We complete the specification of our model by assuming a non-informative prior for the yearly mean infection incidence (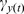) and extra-binomial variation 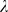 parameters, namely:
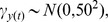


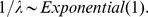



**Table 2:** Informative priors used for the observation model parameters.

	Prior data		Posterior Beta distribution	
Parameter	Successes	Trials	a	b
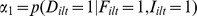	20	51	21	32
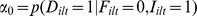	4	108	5	105
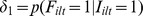	53	168	54	116
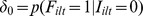	20	931	21	912
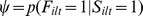	1588	1689	1589	102

Description of the individual-level data (original successes and trials) and the resulting informative prior parameters.

### Model fit

Let 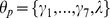 and 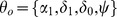 be parameter sets containing the process and observation parameters, respectively. To draw samples from the posterior distribution of our latent states 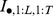 and parameter sets 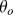 and 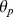, we need to determine 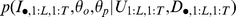
up to a proportionality constant. Our approach adopts a slightly different factorization than the one used in the standard state-space models because the disease dynamics process depends on the observations from the previous time step. Here is our factorization:
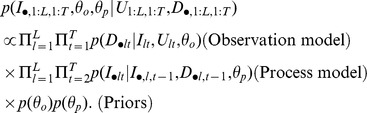



The posterior distribution of the states and parameters 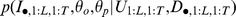
is obtained by Gibbs sampling. We use Metropolis-within-Gibbs sampling steps for all states and parameters due to the lack of a closed form expression for the full conditional distributions. Convergence of our Monte Carlo Markov Chain (MCMC) algorithm was evaluated using trace-plots. All analyses and figures were created using R version 2.13.2 [55].

### Cross-validation exercise

We compare the out-of-sample predictive ability of the proposed model (eqns. 8 and 11) with that of two alternative models. The first model is a phenomenological state-space model, where the latent states follow an AR-1 temporal process, while the second model is a mechanistic Susceptible-Infectious-Susceptible (SIS) model. The goal here is to compare the proposed model to models that would typically be proposed by a statistician (AR-1 process on latent states) or by a mathematical biologist (SIS disease dynamics model). Details regarding the AR-1 and the SIS models are given in Text S1.

To determine the out-of-sample predictive performance of these three models, we conduct a 10-fold cross-validation exercise. First, we randomly partition our dataset into 10 sets. Then, we exclude one of these sets and use our algorithms to predict it based on information from the nine remaining sets. We compare the performance of these models by determining their mean squared error (MSE, a standard model comparison measure that takes into account both bias and variance of estimators), where lower MSE values are preferred.

## Results

### Cross-validation exercise

Our ten-fold cross-validation exercise (i.e., prediction of 10% of the real malaria dataset using the other 90% of the data to train the model) revealed that the proposed model had a consistently better out-of-sample predictive performance when compared to the phenomenological AR-1 state-space model and the mechanistic SIS disease model (Table 3). In particular, the SIS disease model had a substantially worse MSE when compared to the other two models, revealing the negative impact of not allowing for process uncertainty. Based on these cross-validation results, we just report on the results from the proposed model from here onwards.

**Table 3:** The proposed model has better out-of-sample predictive performance than the alternative models.

	MSE		
Subset	Proposed model	AR-1	SIS
1	102	114	273
2	69	77	236
3	97	121	368
4	135	136	280
5	75	88	248
6	88	101	269
7	74	85	286
8	89	105	274
9	90	104	269
10	83	92	258

Mean-squared-error (MSE) for the model proposed in this manuscript (proposed model), the phenomenological state-space model (AR-1), and the mechanistic SIS disease dynamics model (SIS). Data were randomly partitioned into 10 sets and cross-validation results are shown separately for each one of these sets.

**Figure 4:**
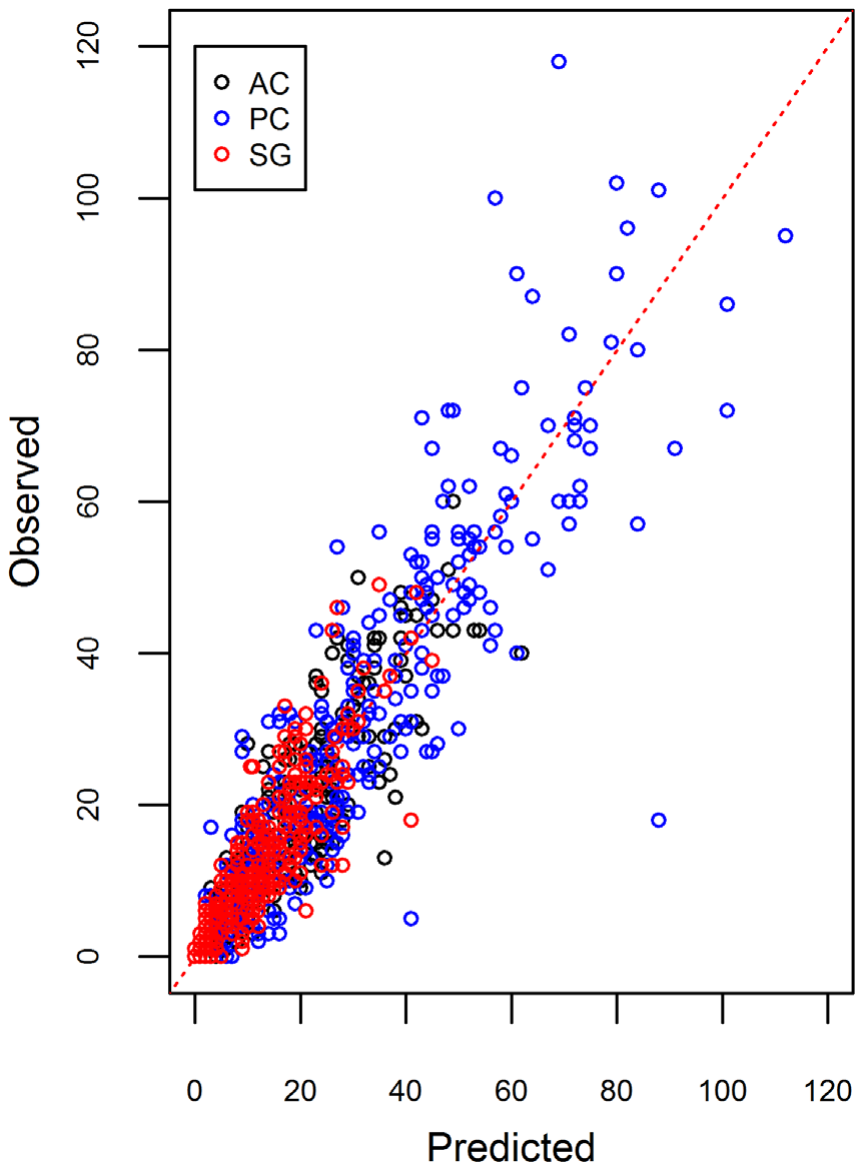
Out-of-sample predictive ability of the proposed model. Comparison of observed vs. predicted number of positive malaria exams. A 1∶1 line was added for reference (dashed red line). Different colors indicate different counties (AC = Acrelandia, PC = Placido de Castro, and SG = Senador Guiomard).

**Figure 5:**
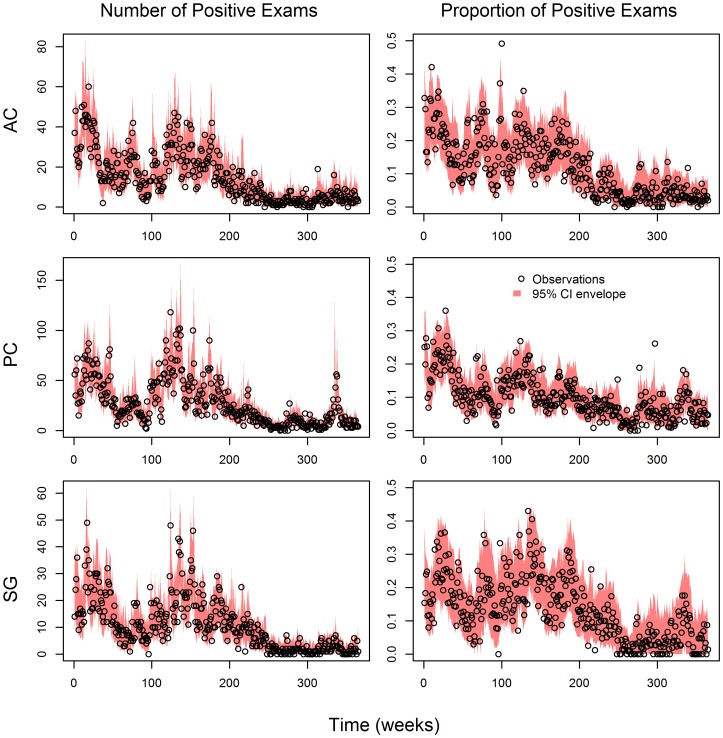
Uncertainty is adequately represented in the proposed model. 95% credible interval (CI) envelopes (red polygons) are overlaid on the data (black circles), both in terms of total number of malaria cases (left panels) and the proportion of positive exams (right panels). Results are displayed separately for each county: Acrelandia (AC, upper panels), Placido de Castro (PC, middle panels), and Senador Guiomard (SG, lower panels).

Using the out-of-sample results, we indeed find that the proposed model fitted well the weekly number of malaria cases (Figure 4). The 95% credible intervals tended to include most of the out-of-sample observations, both in terms of the total number of positive malaria exams (left panels in Figure 5) and the proportion of positive exams (right panels in Figure 5), indicating that uncertainty was adequately represented.

### Simulated data

**Figure 6:**
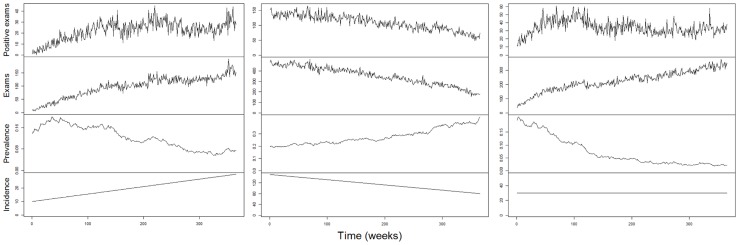
Trends in disease cases do not imply similar trends in infection prevalence or incidence. Number of positive exams, total number of exams, infection prevalence, and infection incidence are depicted from upper to lower panels. Left, middle, and right panels are distinct simulations: infection prevalence decreases but the observed number of disease cases increases (left panels), infection prevalence increases but the observed number of disease cases decreases (middle panels), and trends in infection prevalence and incidence do not match the trend in the observed number of disease cases because of concurrent changes in sampling effort (right panels). Multiple simulations with the same initial infection prevalence showed the same qualitative features.

Simulated data using eqns. 8 and 11 show that trends in the number of malaria cases do not necessarily correspond to equivalent trends in infection prevalence or incidence. For instance, increasing number of malaria cases does not necessarily imply increases in infection prevalence (left panels in Figure 6). Similarly, decreasing number of malaria cases might just reflect decreases in the number of individuals examined, rather than decreases in infection prevalence (middle panels in Figure 6). Finally, trends in the number of malaria cases do not imply similar trend neither in infection prevalence nor in infection incidence (right panels in Figure 6). These simulation results are intuitive if we recognize that the expected number of disease cases depends both on infection prevalence 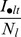 and on the total number of sampled individuals 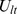 (i.e., 
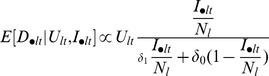
 in eqn. 8). As a consequence, inference on infection prevalence or incidence based solely on the number of positive exams (i.e., ignoring the number of individuals examined) might lead to spurious conclusions.

**Figure 7:**
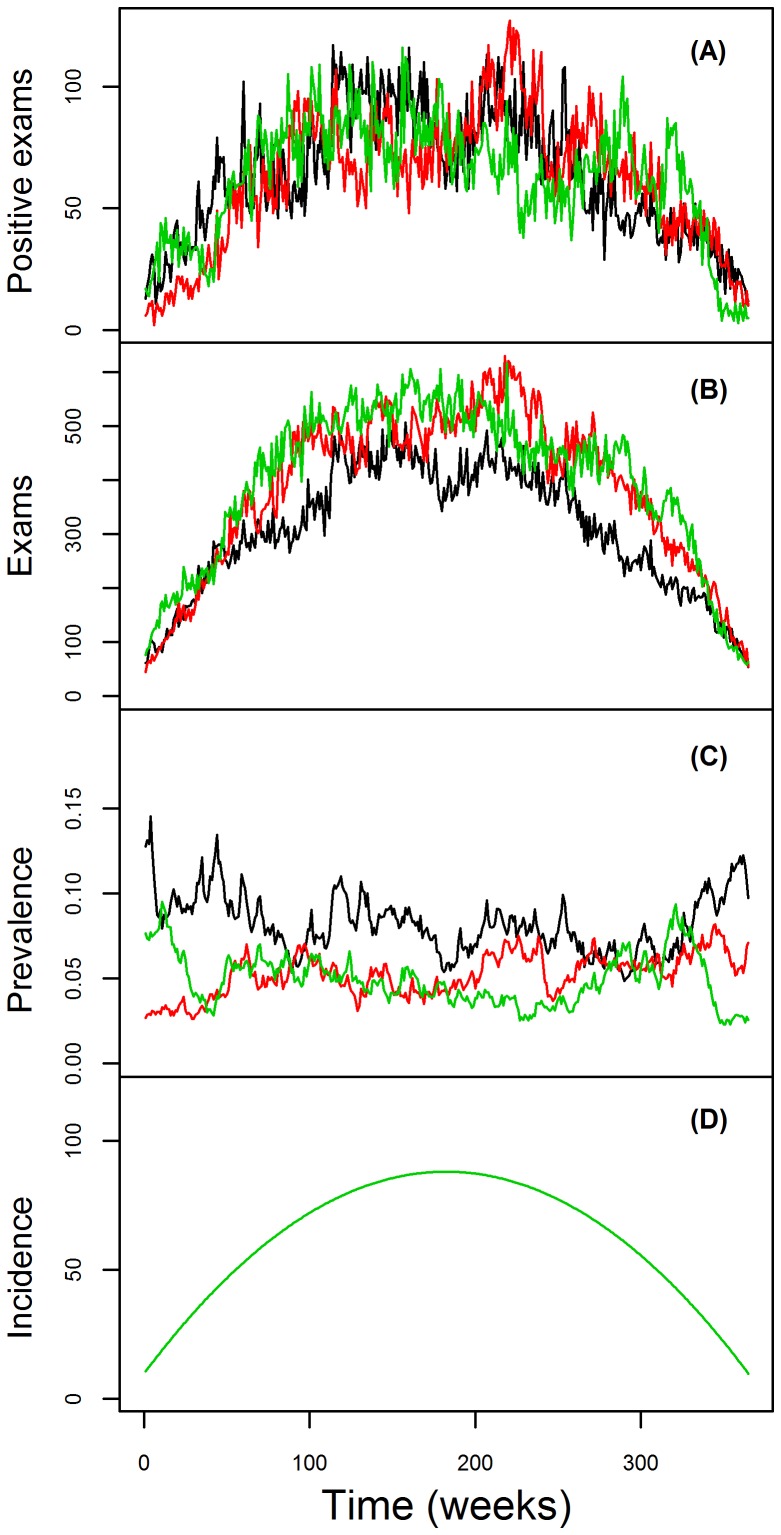
Visual depiction of simulated data. Number of positive exams, total number of exams, infection prevalence, and infection incidence are depicted from upper to lower panels (A–D). Data from the three counties are represented by the black, red, and green lines. Note that, because the true infection incidence is the same for all three counties, the three different lines precisely overlap each other and only the green line appears in panel D.

The importance of allowing observations to directly affect disease dynamics is also illustrated using simulated data. We created a mock dataset where the number of malaria cases, the number of individuals examined, *and* infection incidence all exhibit the same temporal pattern (Panels A, B and D in Figure 7, respectively). As a result of the cancelling effect of greater number of individuals being treated precisely when infection incidence is higher, infection prevalence remains relatively constant (Panels C in Figure 7).

We then estimated infection prevalence and incidence using our original model (eqns. 8 and 10) and compared the resulting inference to that of a similar model that ignores that the observations (i.e., number of treated individuals) decreases infection prevalence. To implement this assumption, we modify equation 10 as
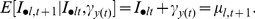
(10a)


**Figure 8:**
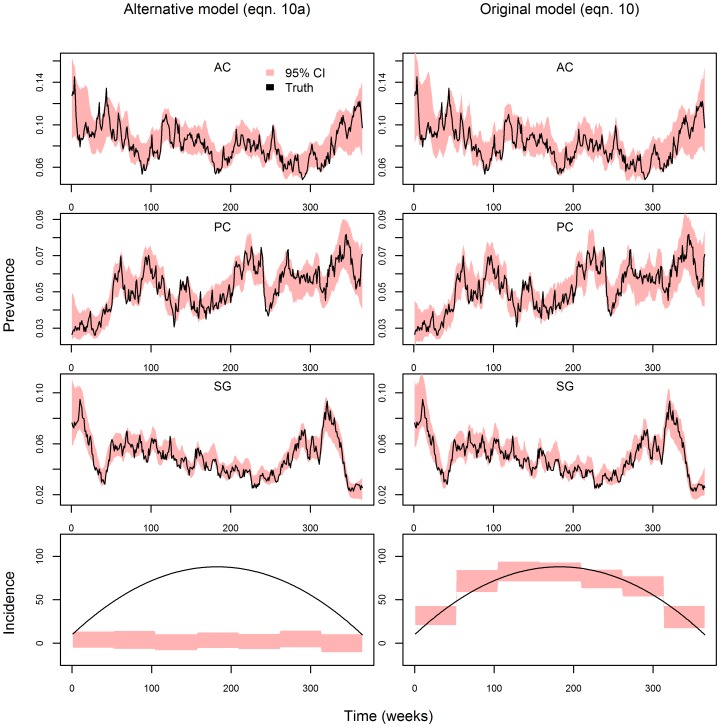
Ignoring the influence of observations on disease dynamics results in misleading inference on infection incidence trends. True infection prevalence for each county (Acrelandia – AC, Placido de Castro – PC, and Senador Guiomard – SG) is depicted in the top six panels (black lines), together with the estimated 95% credible interval for infection prevalence (red polygons). The bottom panels depict the true and inferred infection incidence (black lines and red polygons, respectively). Because simulations and the fitted models assume that the three counties have the same infection incidence, incidence results are displayed in a single panel. Left and right panels show results from the alternative model (eqn. 10a) and original model (eqn. 10), respectively.

Assuming that the observation parameters are known, both the original model and this alternative model inferred well the underlying infection prevalence (top six panels in Figure 8) but led to substantially different inference on infection incidence (bottom two panels in Figure 8). In particular, the original model correctly inferred infection incidence (bottom right panel in Figure 8) while the alternative model inferred an infection incidence of approximately zero (bottom left panel in Figure 8). The intuition for these results is simple. If the number of individuals being treated is changing but the inferred infection prevalence remains constant, this has to imply that the number of individuals being treated is precisely off-setting infection incidence. On the other hand, since the alternative model does not take into account the fact that treated individuals decrease prevalence, an estimated constant infection prevalence implies zero incidence. These results highlight the problem of ignoring that individuals treated for the disease directly influence disease dynamics.

### Case study on malaria

The depiction of the real data in Figure 1 already illustrates that sampling effort exerts considerable influence on the number of positive test results. For instance, the correlation between the number of exams and the number of disease cases was equal to 0.71 in our malaria dataset. Furthermore, there is considerable variation through time in the number of individuals that are examined. Thus, the common assumption that sampling effort is constant is likely to be unrealistic, particularly given the length of many of the disease time-series typically employed, such as those used to detect the effect of climate change on disease. As a result, analyses that rely solely on trends in the number of positive exams may generate misleading conclusions regarding disease dynamics.

**Figure 9:**
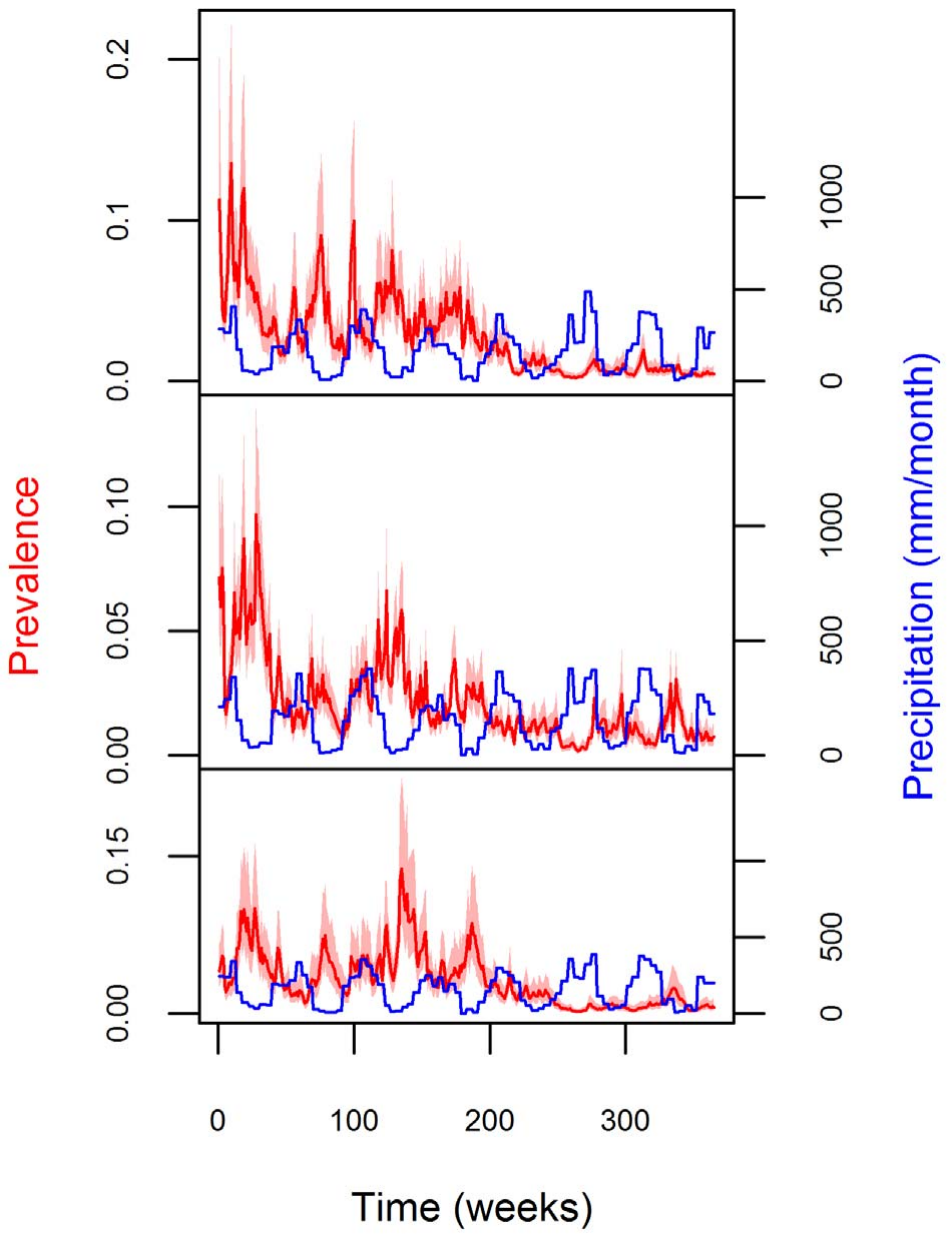
Infection prevalence increases during the dry season. Posterior distribution of infection prevalence (median and 95% credible interval (CI) are depicted as red lines and polygons, respectively, left axes) is compared to monthly precipitation (mm, blue line, right axes). Results are displayed separately for each county: Acrelandia (upper panels), Placido de Castro (middle panels), and Senador Guiomard (lower panels).

Our estimates of infection prevalence reveal a relatively high initial infection prevalence (mean infection prevalence from 2004 to 2008 was 4%, with 95% credible interval (CI) of 3%–5%) with large seasonal outbreaks, which was then followed by a substantial decline in prevalence (mean infection prevalence for 2008–2010 was equal to 0.9% with 95% CI of 0.8–1.1%) (red line and polygon in Figure 9). A large increase in infection incidence seems to occur immediately after the rainy season, leading to subsequent peaks in infection prevalence (which can be as high as 18%) during the dry season, although there is considerable variability both geographically (from county to county) and temporally (year to year). A quantitative measure of association between prevalence and rainfall can be obtained using a permutation test, akin to the ones described in [23]. In this test, we compare precipitation when infection prevalence was at its highest versus at its lowest, for each year and location, yielding 21 (7 years×3 locations) observations for each level of infection prevalence. Our permutation test strongly suggests that the observed difference in mean precipitation is highly unlikely under the null hypothesis of no association (p-value<0.01), consistent with the results from a large-scale analysis of malaria data spanning 7 states of the Brazilian Amazon, which found a negative correlation between precipitation and number of malaria cases [23].

The declining trend in infection prevalence may be attributed to a sharp decrease in incidence after week 210 (from 2007 to 2008, Figure 10); incidence in 2008 to 2010 was approximately 1/5 of the incidence in 2004 to 2007. This abrupt decrease in incidence does not seem to be associated neither with land use/land cover changes (e.g., fire, deforestation rate, and forest cover) nor with climate (e.g., Southern Oscillation index or Oceanic Niño Index) (data not shown). This decrease may be attributable to enhanced vector control activities but we lack data on these activities to test this hypothesis. Posterior distributions for the remaining model parameters are given in Text S1.

**Figure 10:**
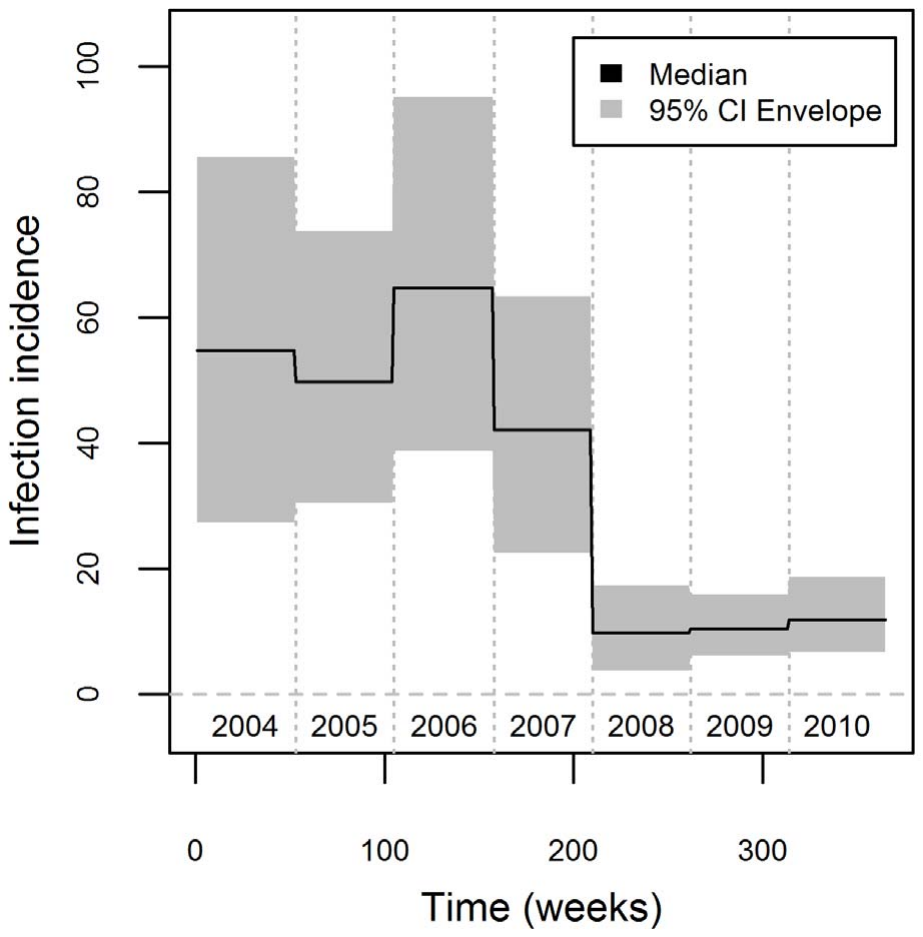
Sharp decrease in infection incidence between 2007 and 2008. Median (black line) and 95% credible interval (grey polygon) for the yearly parameters 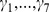. A horizontal line at zero is drawn for reference (grey line) and numbers refer to calendar years.

## Discussion

We have described a novel model that circumvents some of the shortcomings of earlier modeling approaches. For example, our model is able to estimate infection prevalence despite the biases associated with government surveillance data by up-scaling information from a detailed individual level study. This capability of our model is particularly important for public health, where estimates of infection prevalence (rather than disease prevalence) are vital for disease control and elimination strategies. The ability to build on individual-level data (unbiased but geographically limited and costly) to extract information from the government surveillance data (geographically extensive but often biased) is likely to be important for the modeling of data from several other diseases. In particular, it reveals the potential benefits of coordinating careful individual level data collection with the modeling of large-scale patterns using government data. However, for this strategy to work well, it is critical that the collection of individual level data is done so that the results are representative for the region and time-frame of interest.

Disease dynamics model are typically more complex than the model we have presented here, including age structure of the host population, vector dynamics, multiple parasites and strains, and an exposed state. Models containing these additional complexities, however, are rarely fitted to data, with parameters often simply assumed to be known [e.g., 32] or extracted from the literature [e.g.], [ 12], [44,46]. Attempts to fit these models directly to data often reveal that several parameters are unidentifiable [28], [32], [42]–[44], [46] or rely on equilibrium assumptions to estimate these parameters [e.g., 56]. Furthermore, these attempts typically assume either just observation error or just process stochasticity, but not both as our model [50]. Finally, these disease dynamic models have numerous simplifying assumptions of their own, which may lead to substantially different conclusions [47], [48]. For these reasons, we have chosen to employ a model that is not as phenomenological as a regression model or wavelet analysis (i.e., we employ a realistic observation model to infer the underlying infection prevalence and allow for prevalence to decrease with the treatment of individuals) nor mechanistic as disease dynamics models (e.g., we do not account for infection incidence being influenced by current infection prevalence). Cross-validation results suggest that our model may outperform more phenomenological methods (e.g., AR-1 state-space model) and more mechanistic disease models that do not account for process uncertainty (e.g., the deterministic SIS disease dynamics model) (Table 3).

The statistical literature has traditionally assumed that observations do not alter the phenomenon or object that is being measured or assessed. Yet, some types of time-series data can clearly violate this assumption. In our case, a high number of individuals diagnosed to have malaria has the dual-role of suggesting a high infection prevalence at a particular time and a substantial decrease in infection prevalence in the next time step, since these individuals are subsequently treated for the disease. A similar example refers to the use of the number of carcasses encountered or harvested animals as a proxy for animal abundance [57], [58]. The model we propose explicitly accounts for the fact that observations (i.e., the number of individuals diagnosed and then treated for the disease) influence the underlying temporal process (i.e., infection prevalence dynamics), thus modifying the usual state-space approach. Using simulated data, we show that this characteristic is critical when inferring infection incidence (bottom two panels in Figure 8). When applied to the real malaria data, this model characteristic has allowed the identification of pronounced seasonal and long-term trends on infection incidence and prevalence, which might be associated with rainfall. The importance of letting observations affect disease dynamics depends on the nature of the observations. For instance, we believe this is an important problem that has been overlooked in previous malaria models [28], [29]. On the other hand, this feedback of observations on the disease dynamics might not be necessary if the observations consist on the reported number of deaths attributed to a particular disease [e.g.], [ 7,27]. In this case, observations can be modeled simply as a fraction of the true number of individuals that died and left the infected pool.

The proposed model also accounts for sampling effort (i.e., number of individuals sampled), an important characteristic that is surprisingly absent from the disease modeling approaches we know of, mechanistic or not. For example, there has been considerable contention regarding the role of climate change on the increasing number of malaria cases in the African highlands [3], [22], [59]–[61]. Could an increasing trend in sampling effort be a simple explanation for the observed trend in number of malaria cases? Simulated and real data suggest that the effect of sampling effort might be substantial (e.g., Figure 1 and Figure 6), which may be particularly important given the long-term nature of most of the time-series used for disease dynamics modeling [30]. Similar examples highlighting how changes in detection probability and health treatment seeking behavior can distort inference on disease dynamics are also given by [46], [50]. Finally, the lack of more long time-series has been blamed for the considerable uncertainty regarding how climate and other environmental drivers affect disease [29], [30], [62], [63]. Instead of relying on long but rare disease time-series, our model utilized multiple short time-series to infer on the effect of climate on disease dynamics.

In summary, we have focused on three aspects that have typically been ignored by earlier modeling approaches, namely: a) changes in sampling effort (i.e., total number of individuals examined), b) the fact that government surveillance data are often biased towards symptomatic individuals, and; c) the fact that observations (i.e., individuals diagnosed and subsequently treated for the disease) often directly influence disease dynamics by decreasing infection prevalence. We note that the relevance of these aspects fundamentally depends on the particular disease and data that are being analyzed; yet, we highlight them because they (to the best of our knowledge) are overlooked in the literature, either individually or jointly. Furthermore, we emphasize that these shortcomings are not restricted to state-space models; they may occur in other modeling approaches as well. We believe that some of these problems are a legacy from the biomathematical origins of these disease dynamics models. Researchers employing these models have traditionally focused on studying the long-term behavior of this complex non-linear system, thus relying on parameters from the literature or on rough parameter estimates [64]. However, as the focus shifts to parameter estimation and quantitative disease prediction, greater attention will be needed regarding how disease data arise and how to properly estimate parameters from it.

Our modeling approach has five important limitations. First, the proposed model conditions on the total number of exams at each time and county. By doing so, we avoid having to worry about factors that influence the total number of individuals examined, such as the opening of new health facilities, temporary lack of personnel, or shortage of supplies. However, this feature of our model precludes future predictions of future infection prevalence. This limitation can potentially be avoided by creating an additional model to predict the total number of exams. Second, we rely on individual level data to correct for the biased nature of the government surveillance data but individual level data might not be available or might not be representative of the geographical or temporal scale of the aggregate data. In this case, data from the literature might be used in place of the individual level data to create informative priors on the observation model parameters. Third, our observation model assumes that a) symptom status is binary whereas, in reality, there is often a whole spectrum of symptoms [53], which may in turn influence the probability of sampling the individual and detecting the pathogen; and b) that the probability of symptoms given infection 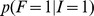 does not change with time. These assumptions may or may not be reasonable for other diseases and we believe that changing our observation model to accommodate for alternative assumptions, without compromising the ability to fit the model, is an important topic for future research.

Fourth, our process model does not take into account the nonlinearities in disease transmission that are the hallmark of disease dynamics models. As noted before, it remains an important challenge to estimate parameter for these biologically inspired disease dynamics models, particularly if one is willing to take into account process uncertainty and a more realistic observation model. Finally, our results suggest large and relatively abrupt changes in infection incidence (Figure 10), which may not be realistic. Future research could focus on developing methods to infer smooth changes in infection incidence.

In this article, we have conceptualized and implemented a model that takes into account how data arise and affect prevalence dynamics. While the exact model formulation (e.g., eqns. 8 and 11) was tailored to the available data and current understanding regarding malaria, the main contribution of this article is to shed light on the importance of a few shortcomings of current disease modeling approaches and to suggest some general strategies to overcome them. We believe that these features have the potential to considerably improve inference on the drivers of disease dynamics when using government surveillance data.

## Supporting Information

Figure S1
**Prior and posterior distributions for yearly infection incidence parameters.** Comparison of the prior (in grey) and posterior distributions (2004 to 2010 in blue to red, respectively) of the yearly infection incidence parameters 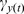.(TIFF)

Figure S2
**Prior and posterior distributions for the extra-binomial variance parameter and observation model parameters.** Comparison of the prior (in grey) and posterior distributions (in black) of the extra-binomial variability parameter 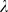 (upper left panel), the probability of symptoms given infected 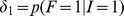 (upper right panel), the probability of not having symptoms given not infected 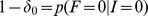 (lower left panel), and the product of the probability of detection given symptoms and infection 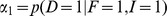 and the probability of having symptoms given that the individual was sampled by the government surveillance network 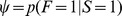 (lower right panel).(TIFF)

Text S1 **Description of the alternative models and posterior distribution of parameters.** Detailed description of the alternative models employed in the validation exercise and posterior distribution of the parameters of the proposed model.
(DOCX)
