# Supplementary figures and images for: Improving the Modeling of Disease Data from the Government Surveillance System: A Case Study on Malaria in the Brazilian Amazon
Source: PLoS Comput Biol. 2013 Nov 7;9(11):e1003312. doi: 10.1371/journal.pcbi.1003312 (PMC3820532; doi:10.1371/journal.pcbi.1003312)

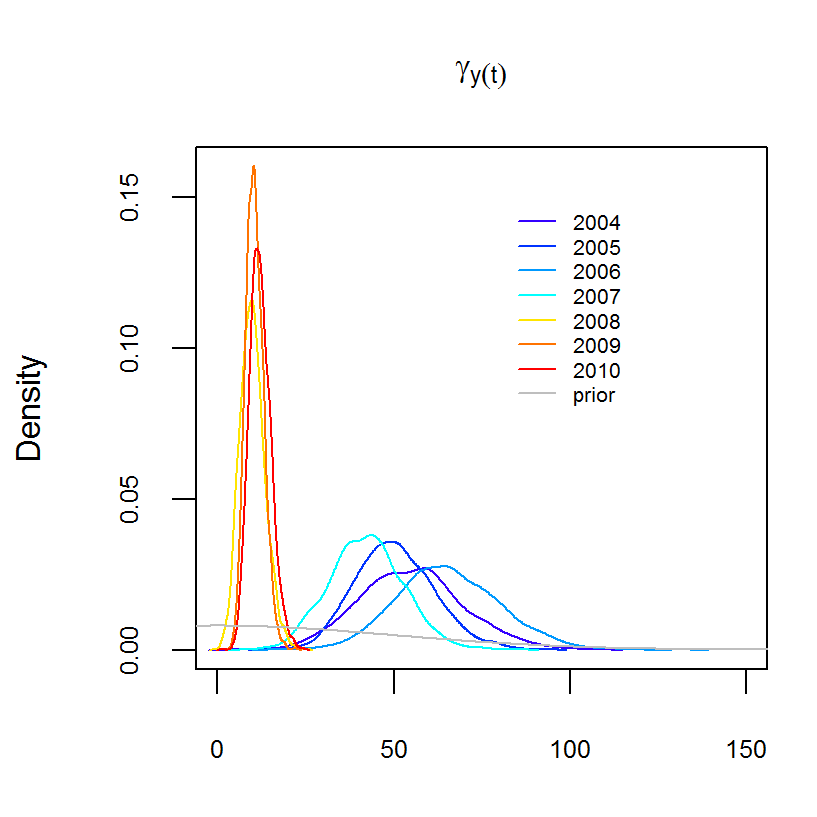

Supplement: Figure S1 — Prior and posterior distributions for yearly infection incidence parameters. Comparison of the prior (in grey) and posterior distributions (2004 to 2010 in blue to red, respectively) of the yearly infection incidence parameters . (TIFF) [file pcbi.1003312.s001.tiff]

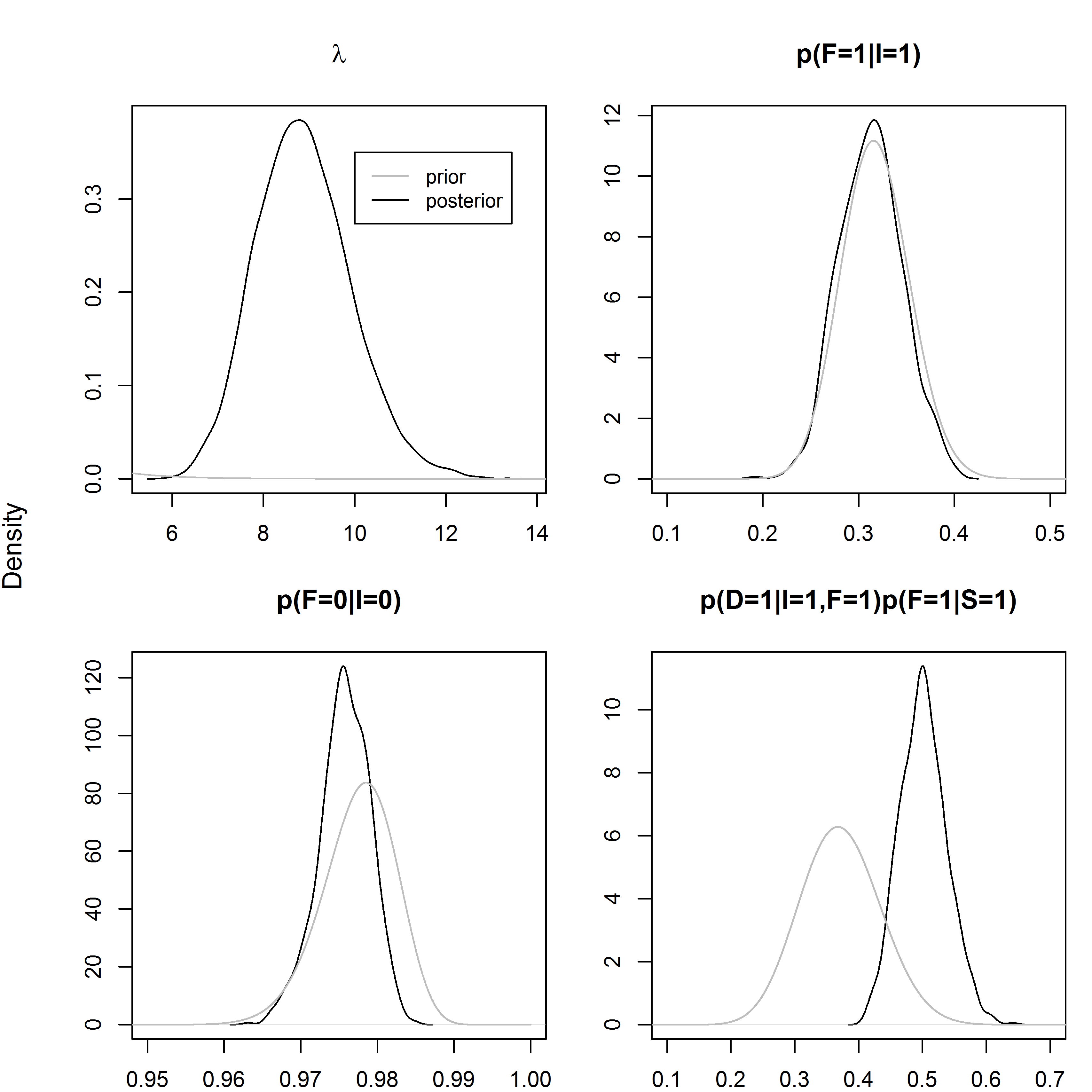

Supplement: Figure S2 — Prior and posterior distributions for the extra-binomial variance parameter and observation model parameters. Comparison of the prior (in grey) and posterior distributions (in black) of the extra-binomial variability parameter (upper left panel), the probability of symptoms given infected (upper right panel), the probability of not having symptoms given not infected (lower left panel), and the product of the probability of detection given symptoms and infection and the probability of having symptoms given that the individual was sampled by the government surveillance network (lower right panel). (TIFF) [file pcbi.1003312.s002.tiff]
