# Supplementary material for: Improving the Modeling of Disease Data from the Government Surveillance System: A Case Study on Malaria in the Brazilian Amazon
Source: PLoS Comput Biol. 2013 Nov 7;9(11):e1003312. doi: 10.1371/journal.pcbi.1003312 (PMC3820532; doi:10.1371/journal.pcbi.1003312)
Supplement: Text S1 — Description of the alternative models and posterior distribution of parameters. Detailed description of the alternative models employed in the validation exercise and posterior distribution of the parameters of the proposed model. (DOCX) [file pcbi.1003312.s003.docx]

# Text S1

## Description of alternative models

Here we describe in detail the two alternative models used in the validation exercise: the phenomenological AR-1 state-space model and the mechanistic SIS model. Both of these models were fitted within a Bayesian framework using a Gibbs sampler, with Metropolis-within-Gibbs sampling steps for parameters for which we did not have closed form expressions for their full conditional distributions. Convergence was assessed by evaluating trace-plots of the parameters and states.

### - Phenomenological AR-1 state-space model

Autoregressive models are central to the analysis of time series data in the statistical literature [1]. Our phenomenological state-space model assumes

, [observation model]

where $z_{lt}$is the latent state. We then assume that these latent states follow an AR-1 process

$\mathbf{z}_{l,1:T}|\eta_{1},...,\eta_{7},\tau,\rho\sim N_{T}(\mathbf{X}_{T\times7}\mathbf{\eta}_{7\times1},\frac{\tau^{2}}{1-\rho^{2}}\mathbf{\Sigma}_{T\times T})$ , [process model]

where the correlation matrix is given by $\mathbf{\Sigma}_{T\times T}=\left[\begin{matrix} 1 & \rho& \rho^{2} & ... \\ \rho& 1 & \rho& ... \\ \rho^{2} & \rho& 1 & ... \\ ... & ... & ... & ... \end{matrix}\right]$. Each row of the design matrix $X_{T\times7}$ is filled with six zeroes and a one in the column that corresponds to the year of the latent state $z_{lt}$ (e.g., a latent state in year 3 has a one in column 3), and $\mathbf{\eta}_{7\times1}$ is a vector containing yearly parameters $\mathbf{\eta}^{T}=[\eta_{1},...,\eta_{7}]$. We adopted vague priors for all parameters, namely

$\eta_{1},...,\eta_{7}\sim N(0,100)$,

$\tau\sim Unif(0,100)$,

$\rho\sim Unif(0,1)$.

Under this specification, we were able to obtain closed form expression for the full conditional distributions of the annual parameters $\mathbf{\eta}$ and the process precision $1/\tau^{2}$. For the remaining parameter $\rho$and latent states ($z_{lt}$), we employ a Metropolis-within-Gibbs sampling steps.

### - Mechanistic SIS disease dynamics model

Disease-dynamics compartment models are extensively used to model disease time series in the mathematical biology literature [2]. One of the simplest disease dynamics model is the Susceptible-Infectious-Susceptible (SIS) model but this model can be easily made more complex by explicitly representing an exposed state, the vector dynamics, and/or different *Plasmodium* species. Here we keep our model simple because problems of parameter identifiability are already evident even in this simple model. Our SIS model assumes that the observed disease incidence arises from a negative binomial distribution with mean $\mu_{lt}$:

$D_{\bullet lt}|n,\mu_{lt}\sim NegativeBinomial\left(n,\frac{n}{n+\mu_{lt}}\right)$, [likelihood]

where the negative-binomial distribution is such that if $x\sim NegativeBinomial(n,p)$ then$E(x)=\frac{n(1-p)}{p}$. This likelihood allows for over-dispersion (described by the parameter *n*) relative to a Poisson distribution with mean $\mu_{lt}$. Notice that we are ignoring sampling effort $U_{lt}$, similar to most mechanistic disease dynamics models. The mean $\mu_{lt}$ is given by:

$\mu_{lt}=\omega\exp(\zeta_{y(t)})I_{_{\bullet lt}}\left(1-\frac{I_{_{\bullet lt}}}{N_{l}}\right)$,

where $\exp(\zeta_{y(t)})I_{_{\bullet lt}}\left(1-\frac{I_{_{\bullet lt}}}{N_{l}}\right)$ is the true infection incidence, $\zeta_{y(t)}$is the yearly parameter governing transmission rate, and $\omega$ is the reporting rate. The underlying deterministic SIS disease dynamics is given by:

$I_{\bullet l,t+1}=I_{\bullet lt}-rI_{\bullet lt}-cI_{\bullet lt}+\exp(\zeta_{y(t)})I_{\bullet lt}(1-\frac{I_{\bullet lt}}{N_{l}})$,

where *r* and *c* are the recovery and cure rates, respectively. This disease dynamics can be slightly simplified to

,

where . We assume vague priors for all parameters () and initial states ($I_{\bullet,1:L,1}$) , namely

$\zeta_{1},...,\zeta_{7}\sim N(0,100)$,

,

$I_{\bullet l1}\sim Unif(0,N_{l})$.

Metropolis-within-Gibbs steps were used to sample the full conditional distributions of these parameters and states.

## Posterior distributions for the parameters in the proposed model

Here we provide a visual comparison of the marginal prior and posterior distributions of all model parameters. This comparison reveals that the priors on the yearly parameters $\gamma_{y(t)}$and $\lambda$ are likely to have had little influence in their corresponding posterior distributions. On the other hand, as expected, the priors on the observation model parameters strongly influenced the corresponding posterior distributions (Figure S1 and Figure S2).

References

1. Prado R, West M (2010) Time series: modeling, computation, and inference: Chapman & Hall/CRC.

2. Keeling MJ, Rohani P (2008) Modeling infectious diseases in humans and animals. Princeton, New Jersey: Princeton University Press.
